# New Medical Journal

**Published:** 1850-03

**Authors:** 


*New Medical Journal.*—We have received the first number of a new Medical Journal, called the *St. Louis Probe,* published at St. Louis, Mo. It is a monthly of 24 pages, edited by A. J. Coons, M.D., and John R. Atkinson, M.D. The title is more novel than attractive. We trust that, like the surgical instrument, the name of which it bears, it may be conducted with skill and discretion. We wish the enterprise all success.

